# Lipopolysaccharide-preconditioned allogeneic adipose-derived stem cells improve erectile function in a rat model of bilateral cavernous nerve injury

**DOI:** 10.1186/s12610-022-00156-w

**Published:** 2022-03-25

**Authors:** Zhenbin Zhang, Pan Nie, Wende Yang, Xiaolei Ma, Zehong Chen, Hongbo Wei

**Affiliations:** Department of Gastrointestinal Surgery, The Third Affiliated Hospital of Sun Yat-sen University, 510630 Guangzhou, China

**Keywords:** Lipopolysaccharide, Adipose-derived stem cells, Cavernous nerve injury, Erectile dysfunction, Corpus cavernosum smooth muscle cells, Fibrosis

## Abstract

**Background:**

Erectile dysfunction (ED) often occurs due to cavernous nerve injury (CNI) after colorectal surgery. Cell-based therapies have great potential for the treatment of CNI-related ED; however, it needs to be optimised. In this study, we explored the therapeutic effects of lipopolysaccharide-preconditioned allogeneic adipose-derived stem cells (L-ADSCs) on CNI-induced ED in rats.

**Results:**

The results of this in vitro study revealed that low-dose lipopolysaccharide could increase the viability of ADSCs, inhibit caspase 3 activation induced by hydrogen peroxide and promote cell migration. Compared with the ADSC supernatant, the L-ADSC supernatant could better reduce fibrosis in the corpus cavernosum smooth muscle cells induced by transforming growth factor-beta 1 protein. In the in vivo study, it was compared to ADSCs therapy, where the L-ADSCs therapy indicated that could better improve erectile function by increasing smooth muscle content and alleviating penile fibrosis in rats 2 weeks after CNI. The outcome may be related to the increase in the hepatocyte growth factor content in the corpus cavernosum and myelin basic protein in the major pelvic ganglion.

**Conclusions:**

L-ADSC treatment may be a promising approach for restoring erectile function after CNI.

**Supplementary Information:**

The online version contains supplementary material available at 10.1186/s12610-022-00156-w.

## Résumé

**Contexte:**

La dysfonction érectile (DE) survient souvent en raison d’une lésion du nerf caverneux après une chirurgie colorectale. Les thérapies cellulaires ont un grand potentiel dans le traitement de la DE liée aux lésions du nerf caverneux; cependant, ces thérapies doivent être optimisées. Dans cette étude, nous avons exploré les effets thérapeutiques des cellules souches adipeuses (ADSC) allogéniques préconditionnées par lipopolysaccharides (L-ADSC) sur la dysfonction érectile induite par lésion du nerf caverneux chez le rat.

**Résultats:**

Les résultats de l’étude in vitro ont révélé que les lipopolysaccharides à faible dose pourraient augmenter la viabilité des cellules souches adipeuses, inhiber l’activation de la caspase 3 induite par le peroxyde d’hydrogène, et favoriser la migration cellulaire. Comparé au surnageant des cellules souches adipeuses, le surnageant des cellules souches préconditionnées (L-ADSC) pourrait mieux réduire la fibrose, dans les cellules musculaires lisses du corps caverneux, induite par la transformation du facteur de croissance-protéine bêta 1. Dans l’étude in vivo, par comparaison au traitement par ADSC, la thérapie par L-ADSC indiquait une meilleure amélioration de la fonction érectile en augmentant le contenu musculaire lisse et en soulageant la fibrose pénienne chez le rat 2 semaines après lésion du nerf caverneux. Le résultat pourrait être lié à l’augmentation de la teneur en facteur de croissance hépatocytaire dans le corps caverneux, et de la protéine de base de la myéline dans le ganglion pelvien majeur.

**Conclusions:**

Le traitement par cellules souches adipeuses (ADSC) allogéniques préconditionnées par lipopolysaccharides (L-ADSC) pourrait être une approche prometteuse pour restaurer la fonction érectile après lésion du nerf caverneux.

## Introduction

Erectile dysfunction (ED) is a common complication that significantly affects the postoperative quality of life in patients with colorectal cancer (CRC). Despite significant advances in surgical techniques, ED may occur in approximately half of the male patients who undergo surgery for CRC [1]. It is mainly attributed to pelvic cavernous nerve injury (CNI), resulting in corporal smooth muscle fibrosis [2, 3]. Phosphodiesterase 5 inhibitor (PDE5I), as the first-line treatment, is not adequately effective in improving this type of ED [4, 5]. Mesenchymal stem cell (MSC)-based therapies have attracted attention owing to their ability to improve ED after CNI [6, 7]. MSCs treatment is considered as a potential therapy because of their paracrine effect, and can be obtained from a variety of sources, such as umbilical cord blood, bone marrow, dental pulp and adipose tissue. Adipose tissue appears to be the ideal source of stem cells because it is abundant and can be harvested by minimally invasive techniques. Compared with other types of stem cells, adipose-derived stem cells (ADSCs) are more active in secreting growth factors and immunomodulators [8, 9]. Moreover, studies have reported that ADSCs are more effective than bone marrow stem cells (BMSCs) in diabetes-related ED and peripheral nerve regeneration [10, 11]. However, when expanded ex vivo and transplanted in vivo, the regenerative and immunomodulatory abilities of MSCs are affected, thereby limiting their clinical application [12]. Thus, it is important to optimise the protection conferred by ADSCs.

Toll-like receptors (TLRs) are chiefly present on immune cells and play a major role in recognizing pathogen-associated molecular patterns in the innate immune system [13]. MSCs also express functional TLRs, which can be confirmed by the response of MSCs to TLR ligands [14]. Lipopolysaccharide (LPS) can be recognized by TLR4, which is present on ADSCs and affects the functions of the cells [15, 16]. The ability of MSCs to produce trophic factors and immunomodulators is enhanced after TLR4 activation [17, 18]. Moreover, LPS can protect MSCs from oxidative stress-induced apoptosis to defend against the harsh environment after transplantation. Some studies revealed that LPS-preconditioned MSCs could accelerate wound healing and improve cardiac function and liver regeneration [19–21]. The underlying mechanism may be that LPS-trained MSCs acquired a type of immune memory to slow down inflammation and fibrosis [22]. Based on the above information, our study aimed to investigate the effects of lipopolysaccharide-preconditioned allogeneic adipose-derived stem cells transplantation on cavernous nerve injury-related erectile dysfunction in rats.

## Materials and methods

### Animals

Ten-week-old male Sprague-Dawley (SD) rats (250–300 g) and four-week-old male SD rats (100 g) were obtained from the Experimental Animal Center of Sun Yat-sen University. All rats were housed in an animal centre. The Institutional Animal Care and Use Subcommittee of the Third Hospital of Sun Yat-sen University approved the present study.

Twenty-four 10-week-old male SD rats were randomly divided into four groups of six rats each. Bilateral CNI (BCNI) was induced by crushing each CN at a distance of 5 mm from the major pelvic ganglion (MPG) for 2 min [6, 7]. Rats in the sham group, wherein BCNI was not induced, underwent laparotomy. The rats in the other three groups were subjected to BCNI and were simultaneously treated with bilateral intracavernous injection of PBS (100 µL; PBS group), ADSCs (1 × 10^6^ ADSCs in 100 µL PBS; ADSCs group) or L-ADSCs (1 × 10^6^ L-ADSCs in 100 µL PBS; L-ADSCs group).

### Cell preparation and identification

ADSCs were isolated from the epididymal adipose tissue of four-week-old male SD rats [23]. Cells were cultured in low-glucose DMEM (Gibco, USA) supplemented with 10% FBS at 37 °C with 5% CO_2_. For LPS preconditioning, the culture medium was replaced by a fresh medium containing 1 µg/mL LPS (O111:B4, Sigma-Aldrich, USA) for 48 h when the ADSCs reached 80% confluence. CD11b, CD29, CD44, CD45 and CD90 antibodies (eBioscience Inc., USA) were used to verify the surface markers of L-ADSCs. The multipotency of L-ADSCs was demonstrated based on osteogenic, adipogenic and chondrogenic differentiation, as described previously [24]. All cells were used at passages 3–7.

Corpus cavernosum smooth muscle cells (CCSMCs) were extracted as described in a previous study [25]. The cavernosal tissues were cut into 1-2 mm pieces and placed in a T25 flask with DMEM supplemented with 10% FBS. The cells migrating from the explants were passaged and purified. CCSMCs were identified by immunofluorescence of an anti-SMA antibody (19245T, 1:500, CST). All cells were used at passages 2–3.

### Cell viability assay

The effect of LPS on the viability of ADSCs was assessed by CCK-8 assay (Dojindo, Japan) following the manufacturer’s protocol. ADSCs were seeded (3 × 10^3^ cells per well) in 96-well plates overnight and subsequently cultured in a fresh medium containing different low concentrations of LPS (0, 0.5, 1, 2, 5 or 10 µg/mL) for 24 or 48 h in a triplicate pattern. We used a microplate reader to measure the absorbance at 450 nm. To test the effect of a high dose of LPS on the viability of ADSCs pretreated with various low doses of LPS at 24 or 48 h, 50 µg/mL LPS was cultured with the cells in 96-well plates for 24 h.

### Cell migration assay

The directed migratory capacities of the cells were evaluated using 8-µm pore membrane filters (BD-353,097, Falcon, USA). Serum-starved ADSCs and L-ADSCs (2 × 10^4^ cells; 100 µL/well) were loaded into the upper chamber. The lower chamber was filled with a serum-free medium. After 24 h, the filter was fixed and stained with 0.1% crystal violet. Cells that had migrated to the lower surface were counted microscopically.

### Apoptosis analysis

ADSCs and L-ADSCs were seeded in six-well plates. To induce apoptosis, ADSCs and L-ADSCs were cultured in a serum-free medium containing H_2_O_2_ at a final concentration of 250 µM for 24 h. Caspase 3 activation in the cells was investigated by western blot analysis.

### Preparation of cell supernatant

ADSCs and L-ADSCs were seeded in T25 flasks and maintained until they reached 90% confluence. Following this, the cells were cultured in a serum-free medium for 48 h. The culture supernatant was collected and stored for subsequent experiments.

### Induction of fibrosis in CCSMCs

TGF-β1 protein (ab236341, USA) was used to induce fibrosis in CCSMCs. CCSMCs were seeded in T25 flasks and maintained until they reached 90% confluence. Following this, the cells were cultured in a serum-free medium or mediums (serum-free medium, ADSC supernatant or L-ADSC supernatant) containing 5 µg/mL TGF-β1 protein for 48 h.

### Erectile function assessment

Changes in intracavernous pressure (ICP) after electrical stimulation of the CN were monitored to assess erectile function in rats 2 weeks after treatment, as previously described [26, 27]. Under anaesthesia with 2.5-3% isoflurane, a heparinized 25-G butterfly needle was inserted into the root of the penis. ICP was recorded using a BL-420 s Biological Functional System (Chengdu Taimeng Technology Ltd., China) by stimulating the CN after exposure. The stimulus parameters were 1.5 mA, 20 Hz and 50 s duration. The mean arterial pressure (MAP) was measured by inserting a heparinized 24-G cannula into the right carotid artery after separation. Erectile function was evaluated based on the ratio of the maximal and total ICP/MAP (area under the curve). Following this, the penises and the MPG were harvested for further assessment.

### PKH67 for cell labelling

ADSCs and L-ADSCs were stained with PKH67 dye (Sigma-Aldrich, USA) according to the manufacturer’s protocol. The cells were injected into the cavernosum of rats with CNI-induced ED. The PKH67 labelled cells in the penis, MPG, lung and spleen were detected using a fluorescence microscope at 3, 7 and 14 days after transplantation.

### Histologic analysis

A portion of the penile tissue and the MPG were collected for immunofluorescent staining as previously described [28]. The penile tissue samples were covered by antibodies SMA (19245T, 1:500, CST) and desmin (5332T, 1:100, CST). The MPG tissue samples were covered by MBP antibodies (AF4085, 1:250, Affinity). Secondary antibodies included DyLight 488- (AB_2556598, 1:500, Invitrogen) and 594- (AB_2556600, 1:500, Invitrogen) conjugated antibodies. Nuclei were stained with DAPI. We obtained the images using a confocal laser scanning microscope (Zeiss LSM 710) or fluorescence microscope (Ni-U, Nikon). The smooth muscle ratio in the penis was evaluated by Masson’s trichrome staining. Quantitative image analysis was implemented using Image-Pro Plus 5.0 (Media Cybernetics, USA).

### Western blot

We lysed the collected penile tissues and cells with RIPA buffer (Beyotime) containing protease inhibitors. A quantity of 40 µg of protein was subjected to electrophoresis using 10% SDS-PAGE and transferred to PVDF membranes. The proteins were probed with antibodies SMA (19245T, 1:1000, CST), desmin (5332T, 1:1000, CST), caspase 3 (AF6311, 1:1000, Affinity), fibronectin (AF0738, 1:1000, Affinity), collagen I (AF7001, 1:750, Affinity), HGF (DF6326, 1:1000, Affinity), TGF-β1 (sc-52,893, 1:500, SANTA CRUZ), BMP 7 (AF5193, 1:1000, Affinity) and GAPDH (AF7021, 1:10000, Affinity). After washing and incubating with secondary antibodies (7074, 1:2000, Abcam), the bands were visualised using an ECL substrate (BL520B, Biosharp).

### Statistical analysis

All data are expressed as means ± standard deviations and analysed using GraphPad Prism (v. 5; GraphPad Software, USA). Statistical analyses were performed using the Student’s t-test in two groups and one-way ANOVA followed by the Tukey-Kramer test for post hoc comparisons between more than two groups. *P* < 0.05 was considered statistically significant.

## Results

### Characterisation of L-ADSCs

**Fig. 1:**
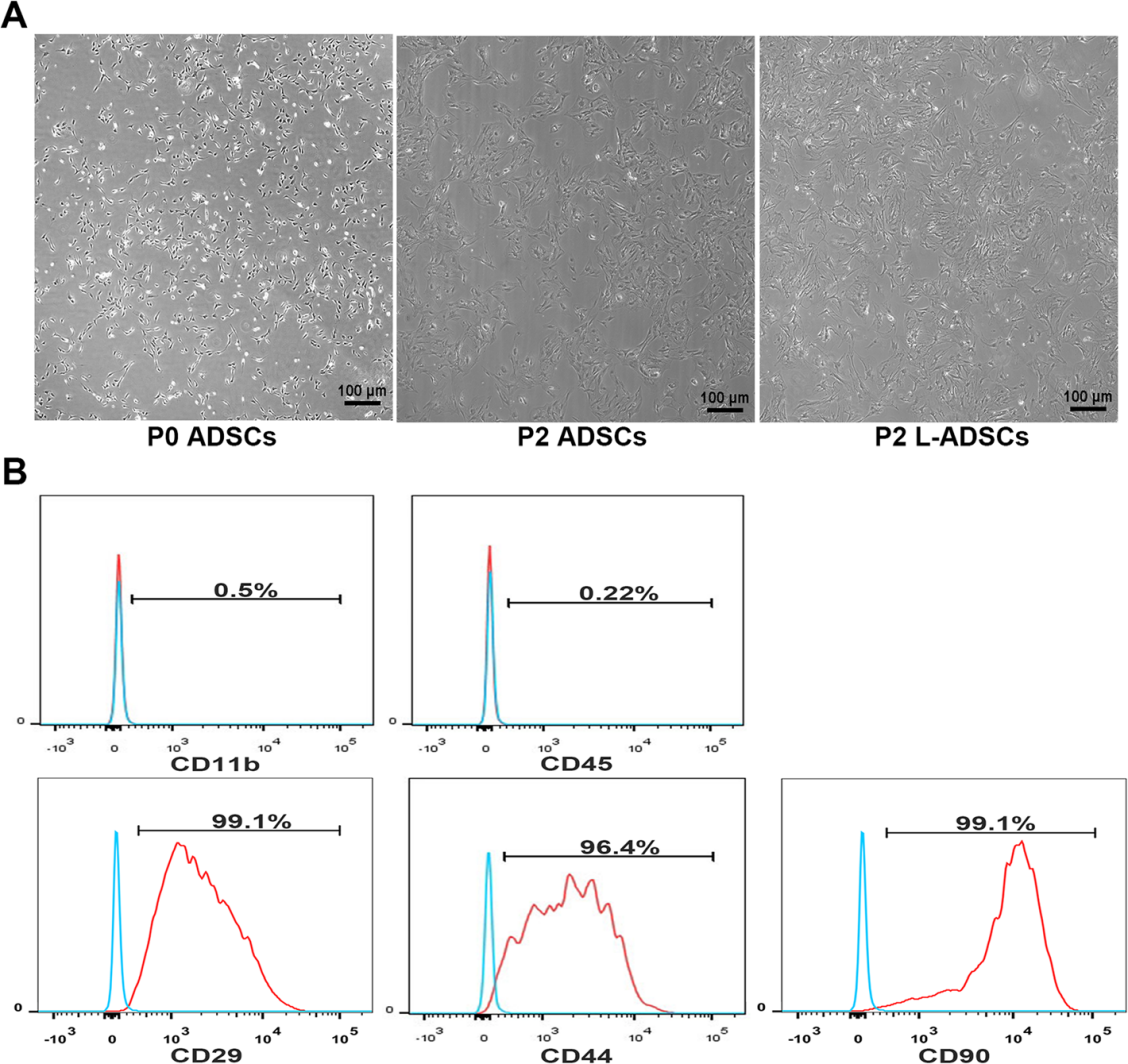
Characterisation of L-ADSCs. **A** Morphology of ADSCs in passage 0 and 2 and L-ADSCs in passage 2. **B** Flow cytometry showing the expression of MSC markers (CD29, CD44 and CD90) but less haematopoietic or endothelial markers (CD11b and CD45) in L-ADSCs. ADSCs: adipose tissue-derived stem cells; L-ADSCs: lipopolysaccharide-preconditioned adipose tissue-derived stem cells

**Fig. 2:**
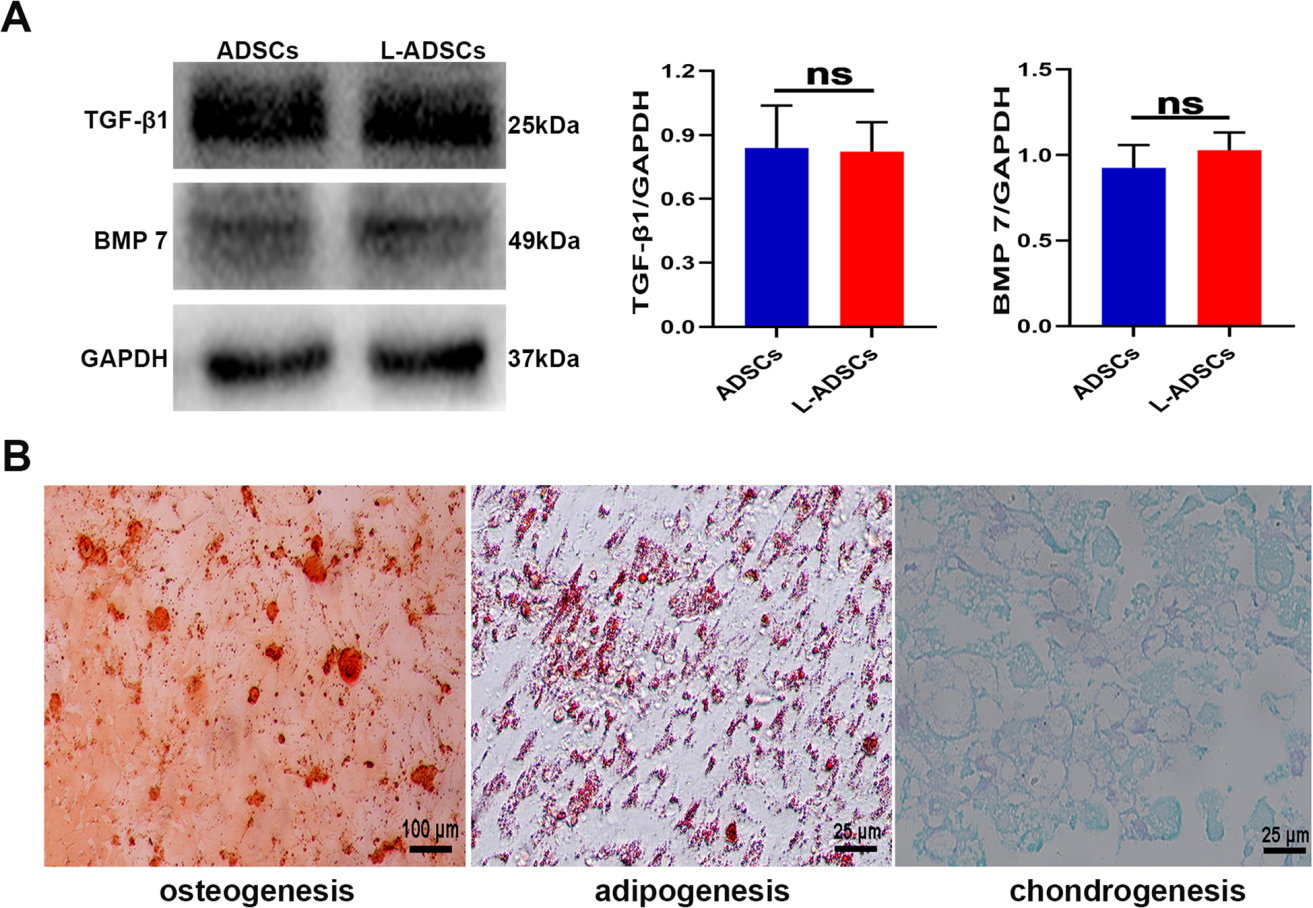
Pluripotency of L-ADSCs. **A** Protein expressions of TGF-β1 and BMP7 were determined by western blot analysis in ADSCs and L-ADSCs. **B** After induction, L-ADSCs demonstrated typical phenotypes of osteocytes (stained with Alizarin Red S), adipocytes (stained with Oil Red O) and chondrocytes (stained with Alcian blue). Error bars: mean ± standard deviation (*n* = 3). ADSCs: adipose tissue-derived stem cells; L-ADSCs: lipopolysaccharide-preconditioned adipose tissue-derived stem cells; TGF-β1: transforming growth factor-beta1; BMP 7: bone morphogenetic protein 7

Primary ADSCs had a typical spindle and fibroblast-like morphology. L-ADSCs exhibited a similar morphology (Fig. 1 A). FACS analysis showed that L-ADSCs expressed mesenchymal markers (CD29, CD44 and CD90) but not endothelial or haematopoietic markers (CD11b and CD45) (Fig. 1B). As shown in Fig. 2 A, protein expressions of TGF-β1 and BMP 7, which play a major role in maintaining the differentiation of MSCs into osteoblasts and chondroblasts, were not significantly different between ADSCs and L-ADSCs. L-ADSCs could differentiate into osteoblasts, adipocytes and chondroblasts (Fig. 2B). L-ADSCs used in this study conformed to the standard criteria for MSCs.

### Effects of LPS on ADSCs

**Fig. 3:**
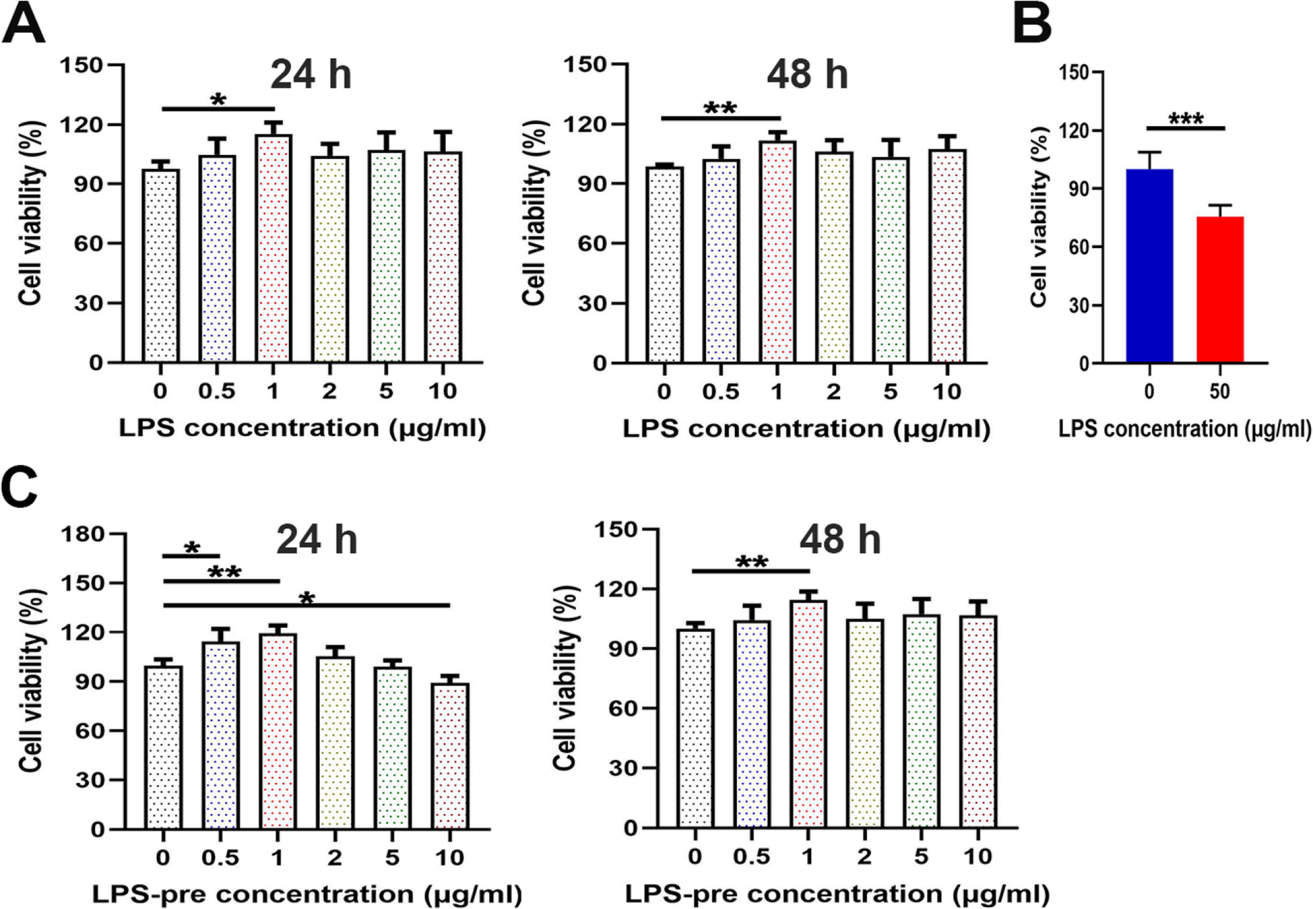
Effects of LPS on ADSC viability. **A** Effects of various low doses of LPS on ADSC viability at 24 and 48 h. **B** Effect of a high dose of LPS (50 µg/mL) on ADSC viability at 24 h. **C** ADSCs pretreated with various low doses of LPS for 24 or 48 h were exposed to a high dose of LPS (50 µg/mL) for 24 h to observe the cellular activity. Error bars: mean ± standard deviation (*n* = 3). *p < 0.05; **p < 0.01; ***p < 0.001. LPS: lipopolysaccharide; ADSCs: adipose tissue-derived stem cells

The CCK-8 assay indicated that exposure to low concentrations of LPS increased the viability of ADSCs at 24 or 48 h. LPS had the most obvious effect on promoting proliferation at 1 µg/mL (*P* < 0.05 at 24 h; *P* < 0.01 at 48 h; Fig. 3 A). Exposure to 50 µg/mL LPS at 24 h significantly reduced the viability of ADSCs (*P* < 0.001; Fig. 3B). To explore the cytoprotective effect of LPS preconditioning on ADSCs, the cells were pretreated with low doses of LPS for 24 or 48 h before being exposed to a high dose of LPS (50 µg/mL). At 1 µg/mL, LPS showed the most effective cytoprotective activity (both *P* < 0.01; Fig. 3 C) and this concentration was selected for subsequent experiments.

**Fig. 4:**
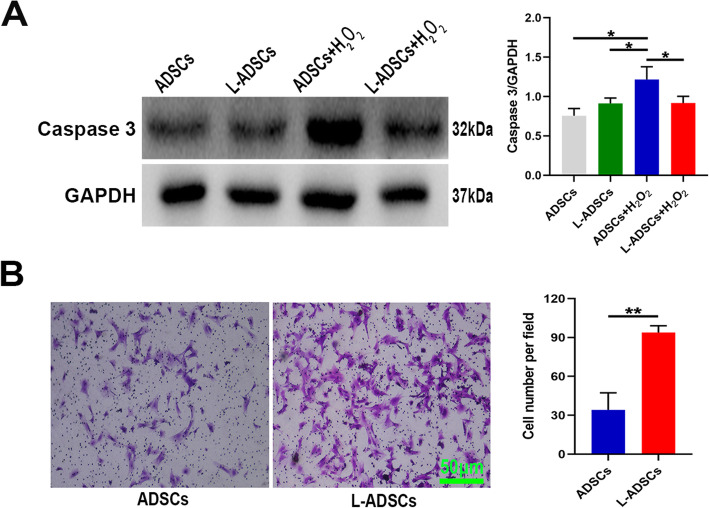
Antiapoptotic and migration capacities of L-ADSCs. **A** Caspase 3 expression in ADSCs and L-ADSCs in the absence or presence of H_2_O_2_ treatment assessed by western blot analysis. **B** Cell number per field of ADSCs and L-ADSCs migrating below the upper chamber assessed by transwell assay. Error bars: mean ± standard deviation (*n* = 3). *p < 0.05; **p < 0.01. ADSCs: adipose tissue-derived stem cells; L-ADSCs: lipopolysaccharide-preconditioned adipose tissue-derived stem cells

The antiapoptotic effects of LPS preconditioning on ADSCs were evaluated. L-ADSCs were capable of inhibiting caspase 3 activation when treated with H_2_O_2_ (*P* < 0.05; Fig. 4 A).

L-ADSCs had a better migration capacity compared with ADSCs (*P* < 0.01; Fig. 4B).

### Fate of the infused cells in rats with BCNI

Labelled ADSCs and L-ADSCs could be detected in the penis, MPG, lung and spleen on Day 3, Day 7 and Day 14 after transplantation in rats with BCNI (Figures S1, S2, S3, S4).

### L-ADSC treatment improved erectile function

**Fig. 5:**
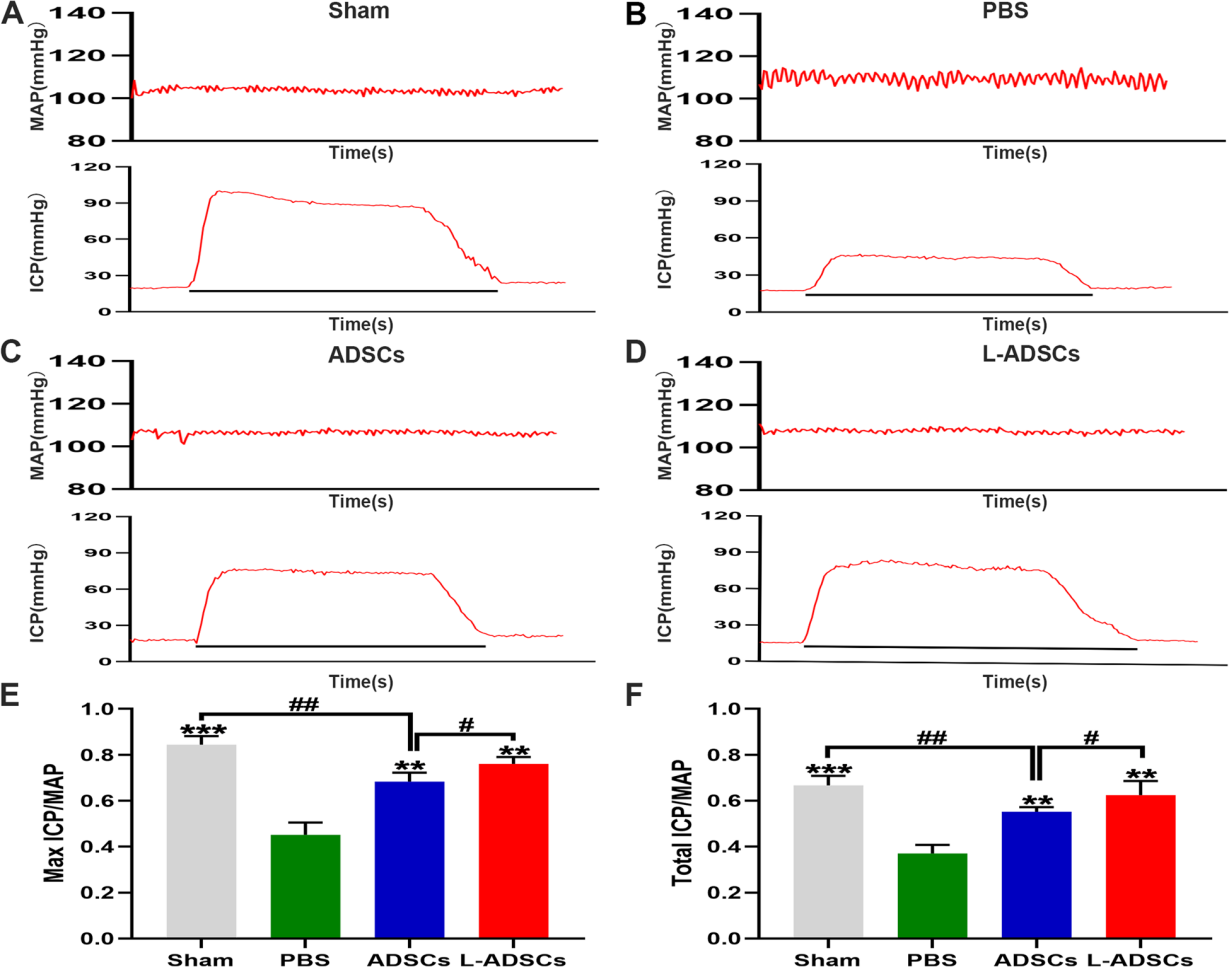
L-ADSC therapy improved erectile function in rats with BCNI. **A-D** The MAP and ICP responded to electrostimulation in the sham, PBS, ADSCs and L-ADSCs groups. The black bar represents the 50 s CN electrical stimulation. **E-F** The ratios of the maximal and total ICP/MAP (area under the curve) in the four groups. Error bars: mean ± standard deviation from *n* = 6 animals per group. **p < 0.01, ***p < 0.001 compared with the PBS group; ^#^*p* < 0.05; ^##^*p* < 0.01 compared with the ADSCs group. ADSCs: adipose tissue-derived stem cells; L-ADSCs: lipopolysaccharide-preconditioned adipose tissue-derived stem cells; BCNI: bilateral cavernous nerve injury; MAP: mean arterial pressure; ICP: intracavernous pressure

ICP and MAP were considered to indicate erectile function through electrical stimulus of the CN. The maximal and total ICP/MAP were significantly lower in the PBS group than that in the sham group (*P* < 0.001; Fig. 5 A-B). Varying degrees of improvements in erectile function were observed in the ADSCs and L-ADSCs group (both *P* < 0.01; Fig. 5 C-D). However, compared with the ADSCs group, the L-ADSCs group demonstrated statistical differences in functional improvement (*P* < 0.05; Fig. 5E F).

### L-ADSC treatment increased the smooth muscle content in the penis

**Fig. 6:**
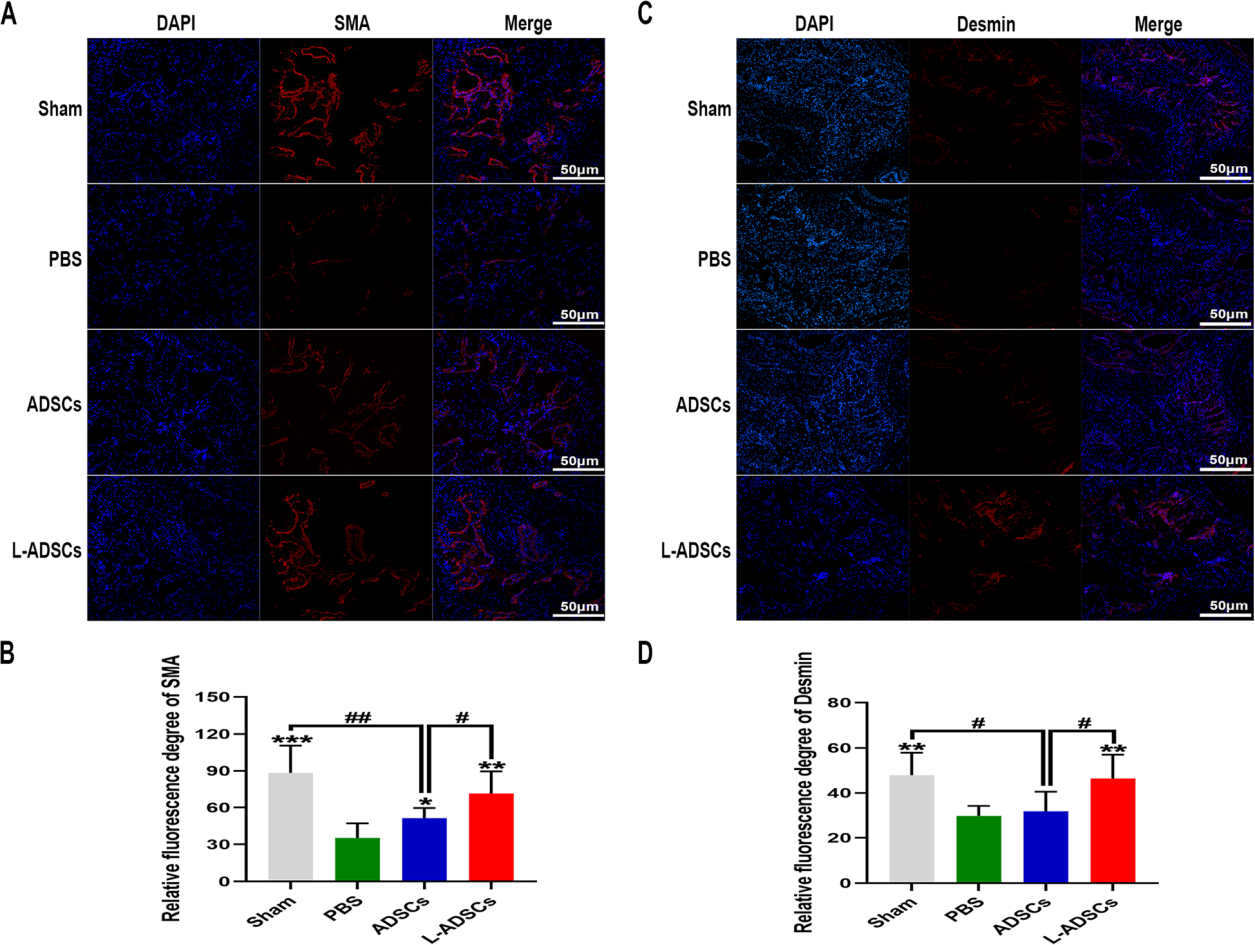
SMA and desmin expressions in the penis by immunofluorescent staining. **A, B** Immunofluorescent staining of SMA in the penis in the sham, PBS, ADSCs and L-ADSCs groups. **C, D** Immunofluorescent staining of desmin in the penis in the four groups. Error bars: mean ± standard deviation from *n* = 6 animals per group. *p < 0.05, **p < 0.01, ***p < 0.001 compared with the PBS group; ^#^*p* < 0.05; ^##^*p* < 0.01 compared with the ADSCs group. ADSCs: adipose tissue-derived stem cells; L-ADSCs: lipopolysaccharide-preconditioned adipose tissue-derived stem cells; SMA: smooth muscle actin

**Fig. 7:**
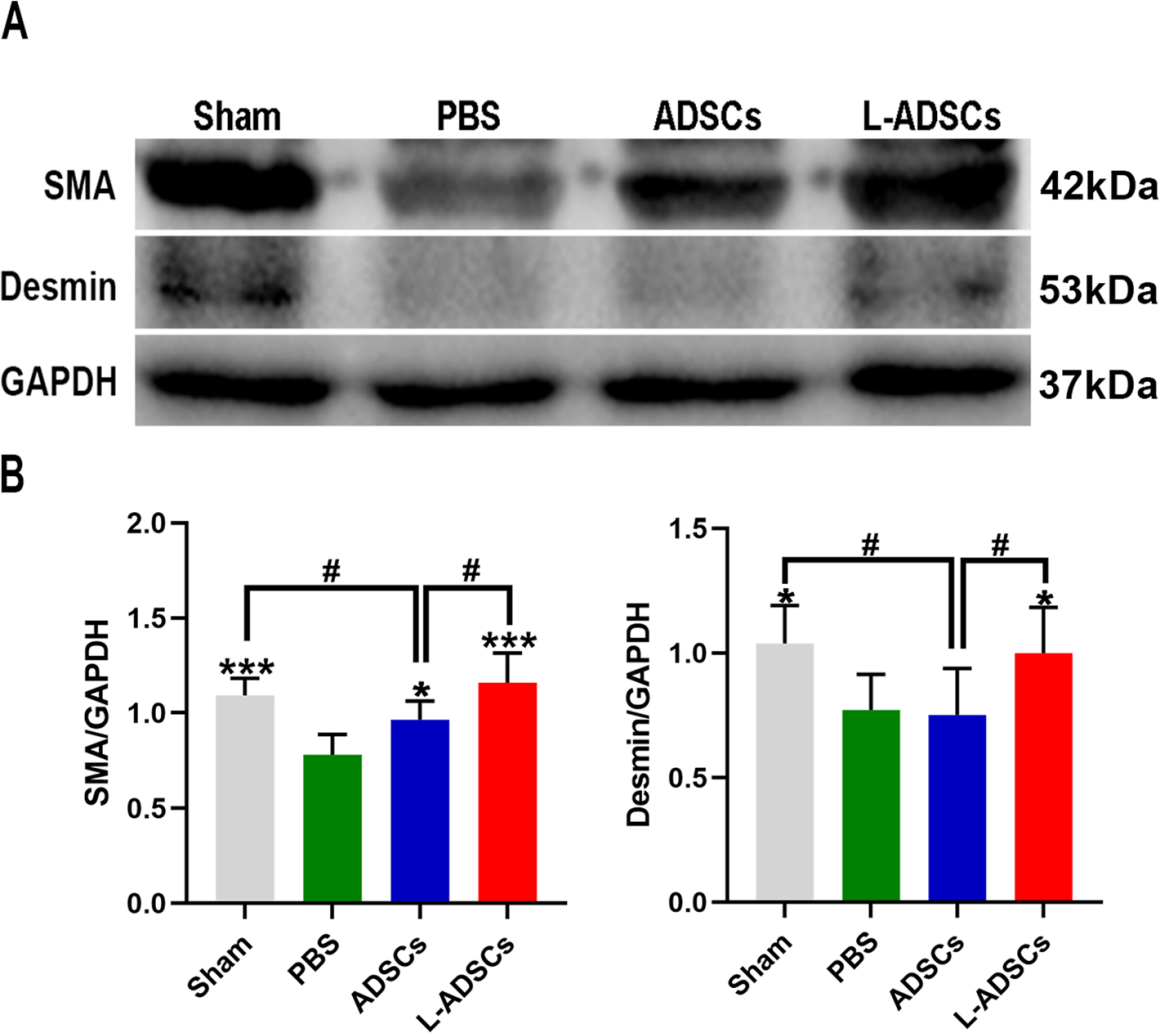
SMA and desmin expressions in the penis by western blot. **A, B** SMA and desmin protein expressions in the penis in the sham, PBS, ADSCs and L-ADSCs groups determined by western blot analysis. Error bars: mean ± standard deviation from *n* = 6 animals per group. *p < 0.05, ***p < 0.001 compared with the PBS group; ^#^*p* < 0.05 compared with the ADSCs group. ADSCs: adipose tissue-derived stem cells; L-ADSCs: lipopolysaccharide-preconditioned adipose tissue-derived stem cells; SMA: smooth muscle actin

**Fig. 8:**
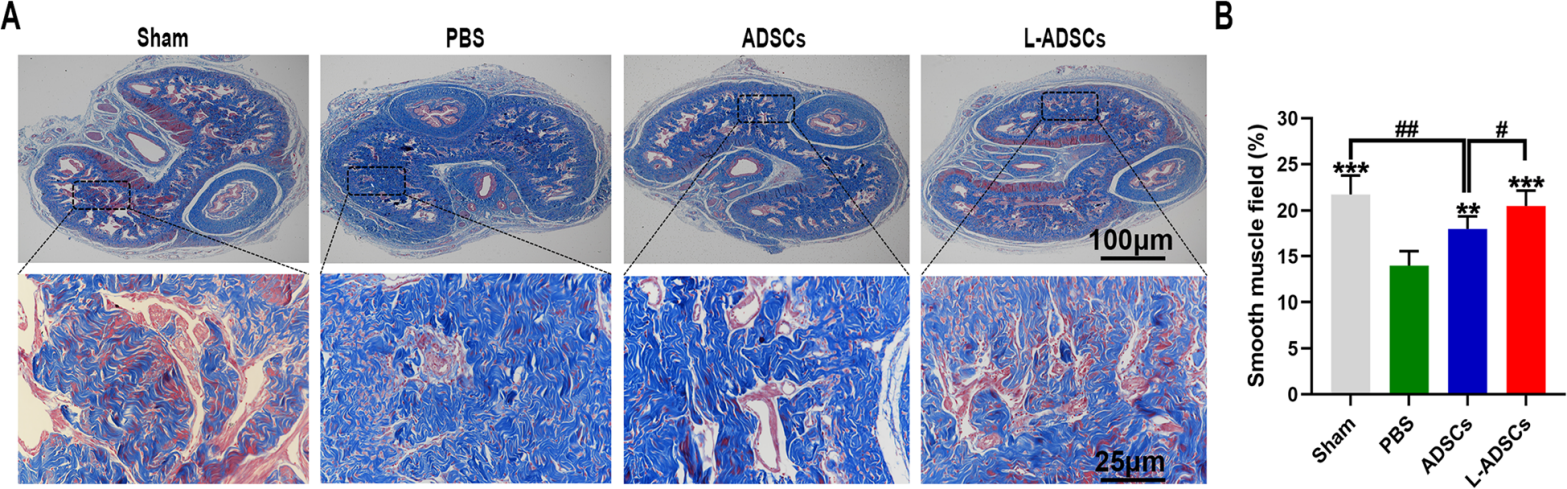
L-ADSC therapy increased the cavernosal smooth muscle content. **A, B** Smooth muscle ratio in the corpus cavernosum assessed by Masson’s trichrome staining in the sham, PBS, ADSCs and L-ADSCs groups. Error bars: mean ± standard deviation from *n* = 6 animals per group. **p < 0.01, ***p < 0.001 compared with the PBS group; ^#^*p* < 0.05; ^##^*p* < 0.01 compared with the ADSCs group. ADSCs: adipose tissue-derived stem cells; L-ADSCs: lipopolysaccharide-preconditioned adipose tissue-derived stem cells

Immunofluorescent staining revealed that the intensity of SMA and desmin in the PBS group was lower than that in the sham group, yet both were rescued by transplantation of L-ADSCs. Transplantation of ADSCs improved the intensity of SMA but not of desmin, compared with the PBS group. Compared with the ADSCs group, the L-ADSCs group showed better improvement (both *P* < 0.05; Fig. 6 A-D). Western blot analysis showed a similar result (Fig. 7 A-B). Masson’s trichrome staining revealed that the smooth muscle ratio was higher in the sham group compared with the PBS group. An increase in the smooth muscle ratio was observed in both the ADSCs and L-ADSCs groups; however, the improvement was better in the L-ADSCs group (*P* < 0.05; Fig. 8 A-B).

### L-ADSC therapy promoted the MBP content in the MPG

**Fig. 9:**
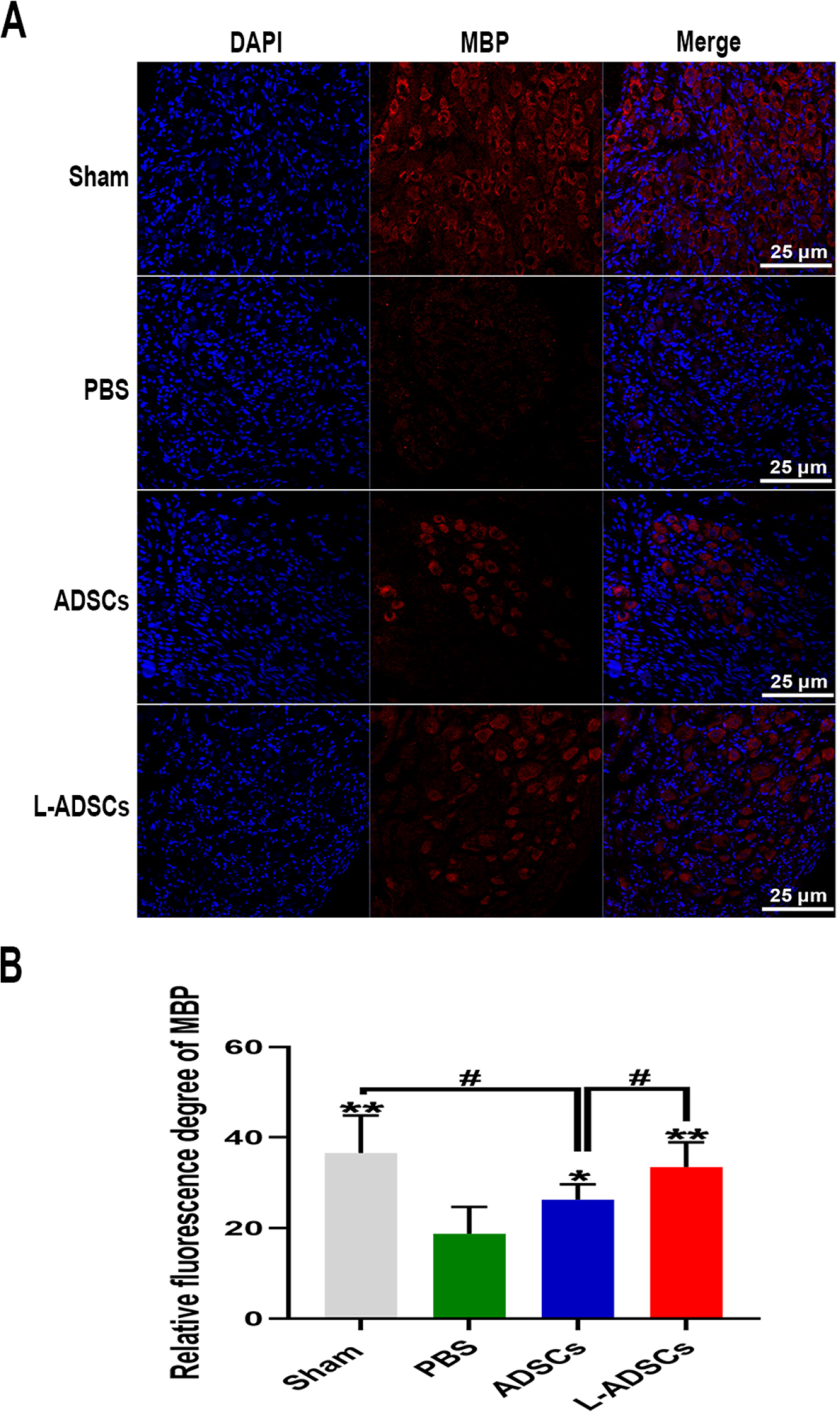
L-ADSC therapy increased the MBP content in the MPG. **A, B** Immunofluorescent staining of MBP in the MPG in the sham, PBS, ADSCs and L-ADSCs groups. Error bars: mean ± standard deviation from *n* = 6 animals per group. *p < 0.05, **p < 0.01 compared with the PBS group; ^#^*p* < 0.05 compared with the ADSCs group. ADSCs: adipose tissue-derived stem cells; L-ADSCs: lipopolysaccharide-preconditioned adipose tissue-derived stem cells; MBP: myelin basic protein; MPG: major pelvic ganglion

Immunofluorescent staining revealed that the expression of MBP in the MPG in the PBS group was lower than that in the sham group, yet it was rescued by transplantation of ADSCs and L-ADSCs. The L-ADSCs group demonstrated better improvement than the ADSCs group (*P* < 0.05; Fig. 9 A-B).

### L-ADSC therapy prevented penile fibrosis by promoting the HGF content in the penis

**Fig. 10:**
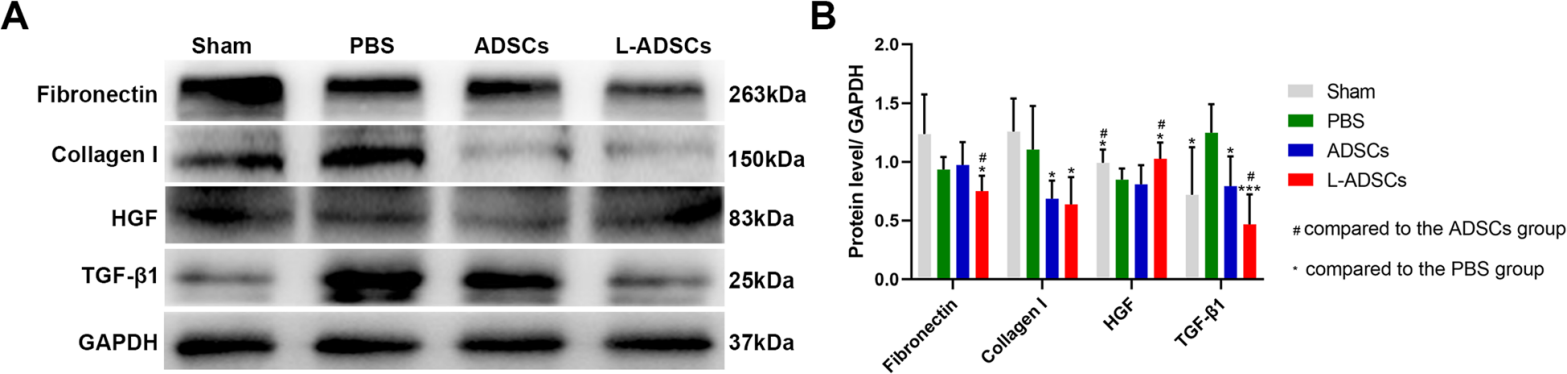
L-ADSCs prevented penile fibrosis. **A, B** Western blot analysis of fibronectin, collagen I, HGF and TGF-β1 protein expressions in penile tissues in the sham, PBS, ADSCs and L-ADSCs groups. Error bars: mean ± standard deviation from *n* = 6 animals per group. *p < 0.05, ***p < 0.001. ^#^*p* < 0.05. ADSCs: adipose tissue-derived stem cells; L-ADSCs: lipopolysaccharide-preconditioned adipose tissue-derived stem cells; TGF-β1: transforming growth factor-beta1; HGF: hepatocyte growth factor

BCNI induced penile fibrosis; however, it was ameliorated by treatment with ADSCs or L-ADSCs. Compared with the ADSCs group, western blot analysis showed that the protein amount of HGF increased, while the expressions of fibronectin and TGF-β1 decreased in the corpus cavernosum in the L-ADSCs group. There was no difference in collagen I expression between the two groups (Fig. 10 A-B).

### L-ADSCs alleviated fibrosis in CCSMCs

**Fig. 11:**
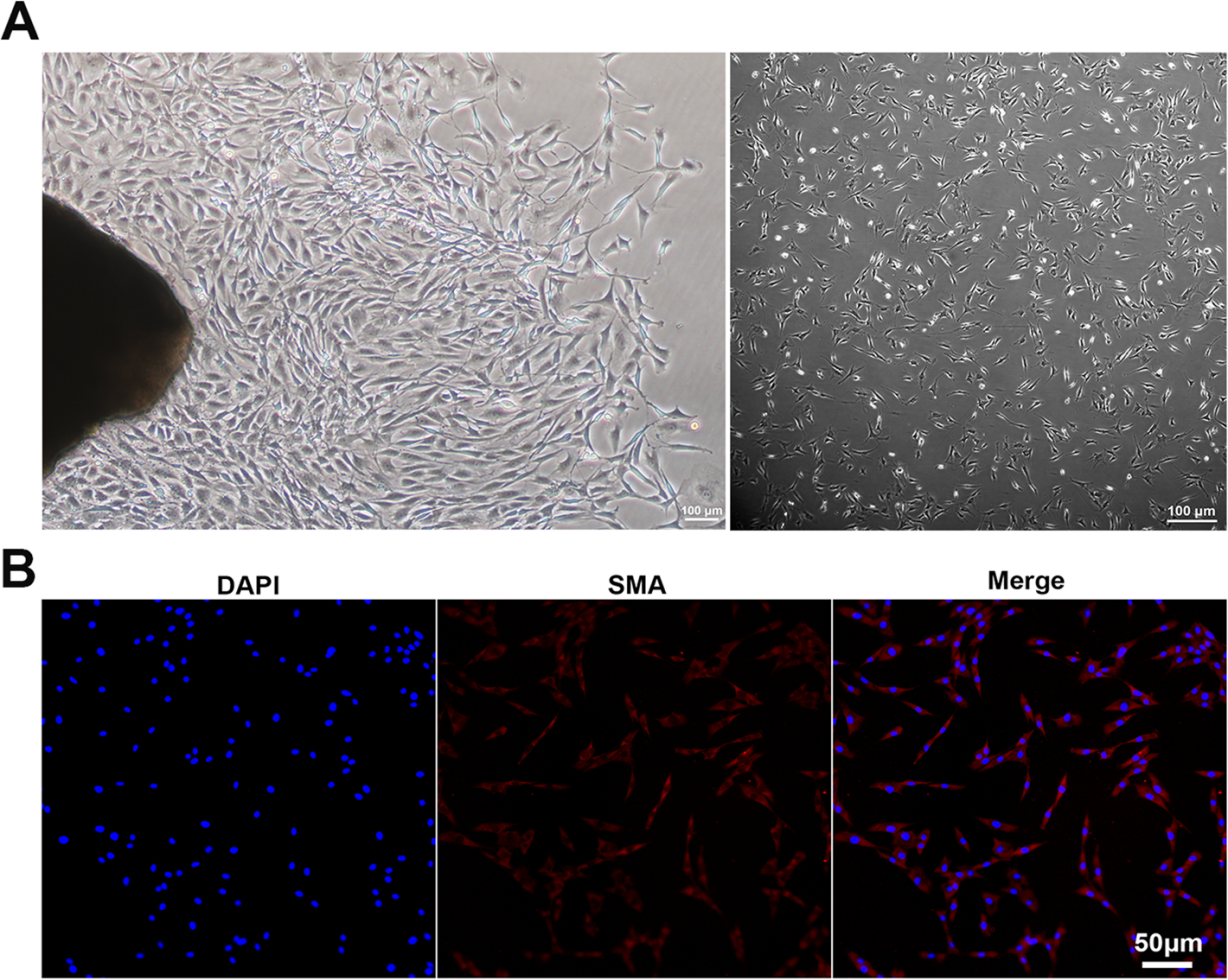
Characterisation of CCSMCs. **A** Primary CCSMCs emerging from the corporal tissue and the passaged cells growing in a whirlpool-like pattern. **B** Immunofluorescence with anti-SMA antibody to identify CCSMCs. CCSMCs: corpus cavernosum smooth muscle cells; SMA: smooth muscle actin

**Fig. 12:**
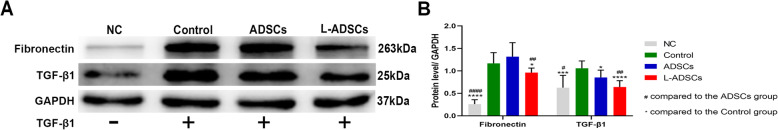
L-ADSCs prevented fibrosis in CCSMCs (**A-B**) Western blot analysis of fibronectin and TGF-β1 protein expressions in CCSMCs cultured in a serum-free medium or mediums (serum-free medium, ADSC supernatant or L-ADSC supernatant) containing 5 µg/mL TGF-β1 protein. Error bars: mean ± standard deviation (*n* = 3). *p < 0.05, ***p < 0.001, ****p < 0.0001. ^#^*p* < 0.05; ^##^*p* < 0.01, ^####^*p* < 0.0001. ADSCs: adipose tissue-derived stem cells; L-ADSCs: lipopolysaccharide-preconditioned adipose tissue-derived stem cells; TGF-β1: transforming growth factor-beta1; CCSMCs: corpus cavernosum smooth muscle cells

CCSMCs migrated from the corpus cavernosum tissues after 3 days, showed a whirlpool-like pattern (Fig. 11 A) and were identified by immunofluorescence labelling of SMA (Fig. 11B). We constructed an in vitro model of CCSMC fibrosis. Western blot analysis indicated that fibrosis in the CCSMCs could be induced by adding TGF-β1 protein to the medium; however, this could be alleviated by ADSC or L-ADSC supernatant. As shown in Fig. 12 A-B, compared with the ADSCs supernatant group, the protein amounts of fibronectin and TGF-β1 in CCSMCs were decreased in the L-ADSC supernatant group (both *P* < 0.01).

## Discussion

ED is a frequent complication that affects the physical and psychosocial health of male patients after surgery for CRC. Although pelvic autonomic nerve preservation surgery is performed, the rate of ED remains high [29, 30]. PDE5I, as the first-line therapy, does not provide satisfactory results [31, 32]. MSC-based therapy has been shown to be effective; however, it needs to be optimised before clinical application. Therefore, the efficacy of L-ADSC therapy was investigated in this study. We found that treatment with L-ADSCs improved erectile function better by increasing the smooth muscle content in the penis and MBP content in the MPG and preventing fibrosis of penile tissues.

The rapid apoptosis and low survival of MSCs following transplantation may limit their therapeutic application. Therefore, our interest lies in enhancing the potency of MSCs. Cell preconditioning might be a powerful strategy to tackle this problem. Studies have demonstrated that MSCs can sense their environment and respond adaptively [33, 34]. We investigated the effect of LPS preconditioning on ADSCs in this study. Our results revealed that a low dose of LPS could promote the proliferation of ADSCs in vitro. Herzmann et al. established that LPS induced the proliferation of human ADSCs via TLR4 activation [15]. However, Xu et al. reported that LPS did not have any significant influence on the viability of mouse BMSCs [35]. The differences may be associated with LPS from different E. coli strains or MSCs from different tissues. Meanwhile, we observed that a low-dose LPS pretreatment protected ADSCs from oxidative stress in vitro, which is beneficial to improve the efficacy of cell transplantation.

MBP is an important protein involved in the myelination of peripheral nerve cells. Loss of MBP would lead to a decrease in the axonal length and abnormal Schwann cells-axon contact. In our study, L-ADSC therapy increased the expression of MBP in the MPG in rats with BCNI, which helped repair the cavernous nerve axons and promoted the recovery of erectile function.

BCNI can cause irreversible progression of penile tissue fibrosis, which occurs early after injury through dynamic deposition and degradation of the extracellular matrix (ECM). A study showed that smooth muscle decreased and fibrotic collagen increased 1 year after radical prostatectomy compared with preoperative biopsies [36]. Wan et al. reported that inhibition of lysyl oxidase could prevent penile fibrosis by decreasing the ECM (collagen I/IV and fibronectin) protein expression in the corpus cavernosum after BCNI [37]. Wu et al. reported that transplantation of human gingiva-derived MSCs could inhibit the ECM (collagen I/IV and fibronectin) protein expression in the corpus cavernosum in rats with BCNI [38]. The expressions of collagen I and fibronectin were investigated in our study. Compared with the PBS and ADSCs groups, our in vivo results revealed decreased fibronectin expression in the corpus cavernosum in the L-ADSCs group. However, L-ADSC therapy could not further reduce the expression of collagen I in the penis compared with ADSC therapy. However, the results of both groups were superior to those observed in the PBS group.

TGF-β functions in all types of fibrosis and is a potent stimulator of ECM protein synthesis. Jin et al. reported that the expression of TGF-β1 increased after CNI in a mouse model [39]. Furthermore, Nehra et al. demonstrated that TGF-β1 could induce fibrosis in the corpus cavernosum of rabbits in vivo [40]. Thus, TGF-β1 is a good marker as a pro-fibrotic cytokine in the penis after BCNI. Our study proved that, compared with the PBS and ADSCs groups, TGF-β1 expression decreased in the L-ADSCs group in vivo.

The effects of L-ADSCs on the production of HGF in the corpus cavernosum were evaluated to explore its potential mechanism. HGF is a pleiotropic factor that has anti-fibrotic and pro-regenerative effects. In our study, HGF levels were increased in penises of rats in the L-ADSCs group, which might be the one reason why L-ADSCs restored erectile function better. Das et al. demonstrated that injections of rh-HGF protein significantly improved erectile function in diabetic mice [41]. Liu et al. reported that a combination of HGF and ADSCs improved erectile function in diabetic rats by down-regulating TGF-β1-Smad signaling [42].

In addition, we constructed a model of CCSMC fibrosis, which has not been reported previously. Compared with the control and ADSC supernatant groups, the protein expressions of fibronectin and TGF-β1 in CCSMCs were lower than those in the L-ADSC supernatant group. We believe this model is beneficial to explore fibrosis in CCSMCs in future in vitro studies.

Our study has some limitations. First, the results obtained using a rat model may not necessarily apply to humans. Second, the underlying mechanisms of HGF functioning in BCNI-induced ED need to be fully addressed.

## Conclusions

The present study showed that L-ADSCs improved erectile function more effectively in rats after BCNI by increasing the smooth muscle content in the penis and MBP in the MPG and preventing fibrosis of penile tissues. The improvement might be related to the increase in the HGF content in the corpus cavernosum and the promotion of nerve regeneration in the MPG. Therefore, L-ADSC therapy could be a promising approach for the treatment of BCNI-induced ED.

## Supplementary information

Additional file 1 **Figure S1. **PKH67 labelled cells in the penis. **A-B** Labelled ADSCs and L-ADSCs could be detected in the penis on Day 3, Day 7 and Day 14 after transplantation in rats with BCNI. ADSCs: adipose tissue-derived stem cells; L-ADSCs: lipopolysaccharide-preconditioned adipose tissue-derived stem cells; BCNI: bilateral cavernous nerve injury.

Additional file 2 **Figure S2. **PKH67 labelled cells in the MPG. **A-B** Labelled ADSCs and L-ADSCs could be detected in the MPG on Day 3, Day 7 and Day 14 after transplantation in rats with BCNI. ADSCs: adipose tissue-derived stem cells; L-ADSCs: lipopolysaccharide-preconditioned adipose tissue-derived stem cells; BCNI: bilateral cavernous nerve injury; MPG: major pelvic ganglion.

Additional file 3 **Figure S3. **PKH67 labelled cells in the lung. **A-B** Labelled ADSCs and L-ADSCs could be detected in the lung on Day 3, Day 7 and Day 14 after transplantation in rats with BCNI. ADSCs: adipose tissue-derived stem cells; L-ADSCs: lipopolysaccharide-preconditioned adipose tissue-derived stem cells; BCNI: bilateral cavernous nerve injury.

Additional file 4 **Figure S4. **PKH67 labelled cells in the spleen. **A-B** Labelled ADSCs and L-ADSCs could be detected in the spleen on Day 3, Day 7 and Day 14 after transplantation in rats with BCNI. ADSCs: adipose tissue-derived stem cells; L-ADSCs: lipopolysaccharide-preconditioned adipose tissue-derived stem cells; BCNI: bilateral cavernous nerve injury.

## Data Availability

The datasets used and/or analysed during the current study are available from the corresponding author on reasonable request.

## Abbreviations

ADSCs: Adipose-derived stem cells
BCNI: Bilateral cavernous nerve injury
BMP 7: Bone morphogenetic protein 7
BMSCs: Bone marrow stem cells
CCK-8: Cell Counting Kit-8
CCSMCs: Corpus cavernosum smooth muscle cells
CNI: Cavernous nerve injury
CRC: Colorectal cancer
DMEM: Dulbecco’s modified eagle medium
ECM: Extracellular matrix
ED: Erectile dysfunction
FBS: Fetal bovine serum
GAPDH: Glyceraldehyde-3-phosphate dehydrogenase
H_2_O_2_: Hydrogen peroxide
HGF: Hepatocyte growth factor
ICP: Intracavernous pressure
L-ADSCs: Lipopolysaccharide-preconditioned adipose-derived stem cells
LPS: Lipopolysaccharide
MAP: Mean arterial pressure
MBP: Myelin basic protein
MPG: Major pelvic ganglion
MSCs: Mesenchymal stem cells
PDE5I: Phosphodiesterase 5 inhibitor
PVDF: Polyvinylidene fluoride
SD: Sprague-Dawley
SDS-PAGE: Sodium dodecyl sulfate-polyacrylamide gel electrophoresis
SMA: Smooth muscle actin
TGF-β1: Transforming growth factor-beta1
TLRs: Toll-like receptors
