# Supplementary figures and images for: Lipopolysaccharide-preconditioned allogeneic adipose-derived stem cells improve erectile function in a rat model of bilateral cavernous nerve injury
Source: Basic Clin Androl. 2022 Mar 25;32:5. doi: 10.1186/s12610-022-00156-w (PMC8953072; doi:10.1186/s12610-022-00156-w)

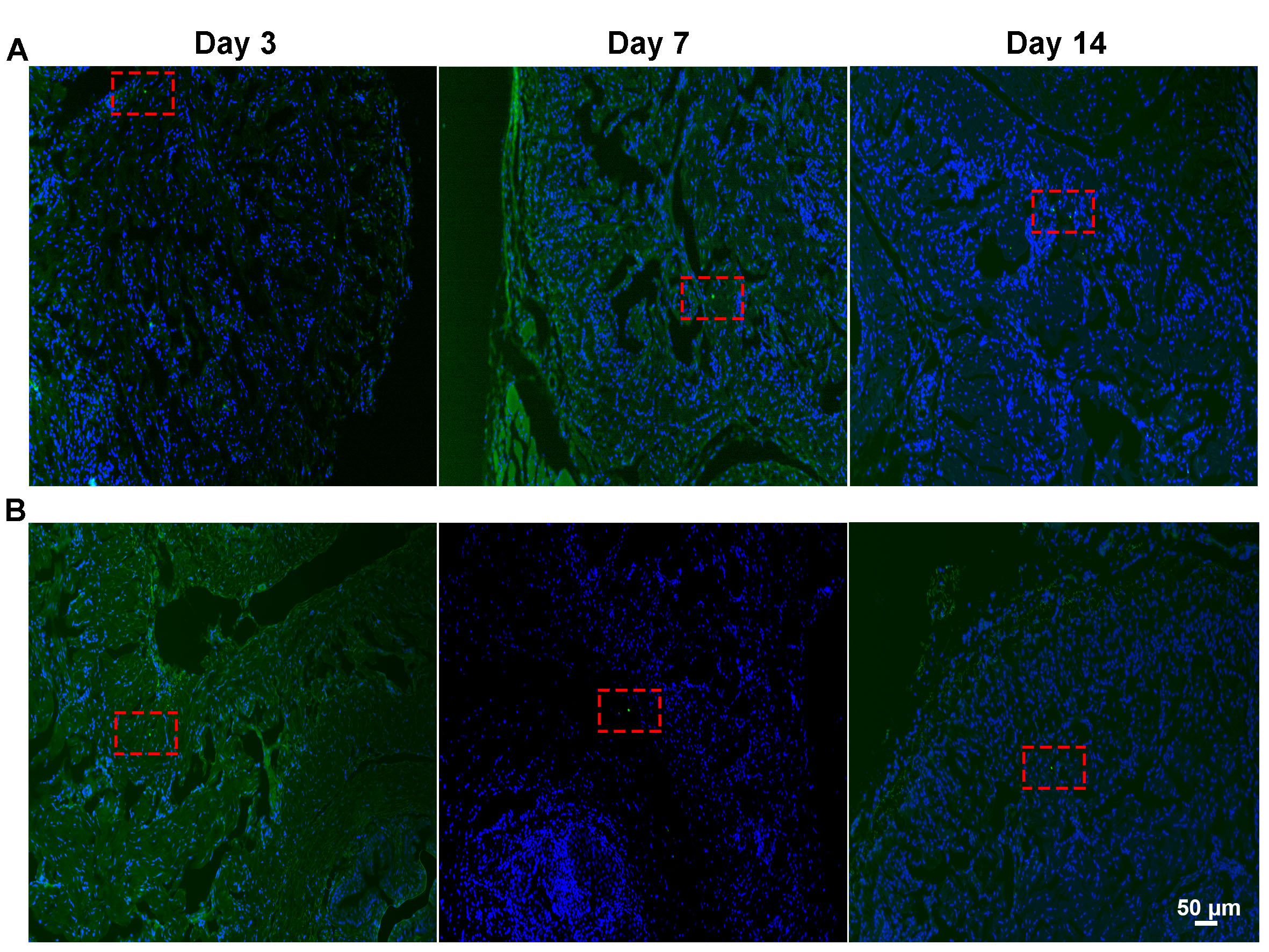

Supplement: Supplementary file 1 — Figure S1. PKH67 labelled cells in the penis. A-B Labelled ADSCs and L-ADSCs could be detected in the penis on Day 3, Day 7 and Day 14 after transplantation in rats with BCNI. ADSCs: adipose tissue-derived stem cells; L-ADSCs: lipopolysaccharide-preconditioned adipose tissue-derived stem cells; BCNI: bilateral cavernous nerve injury. [file 12610_2022_156_MOESM1_ESM.jpg]

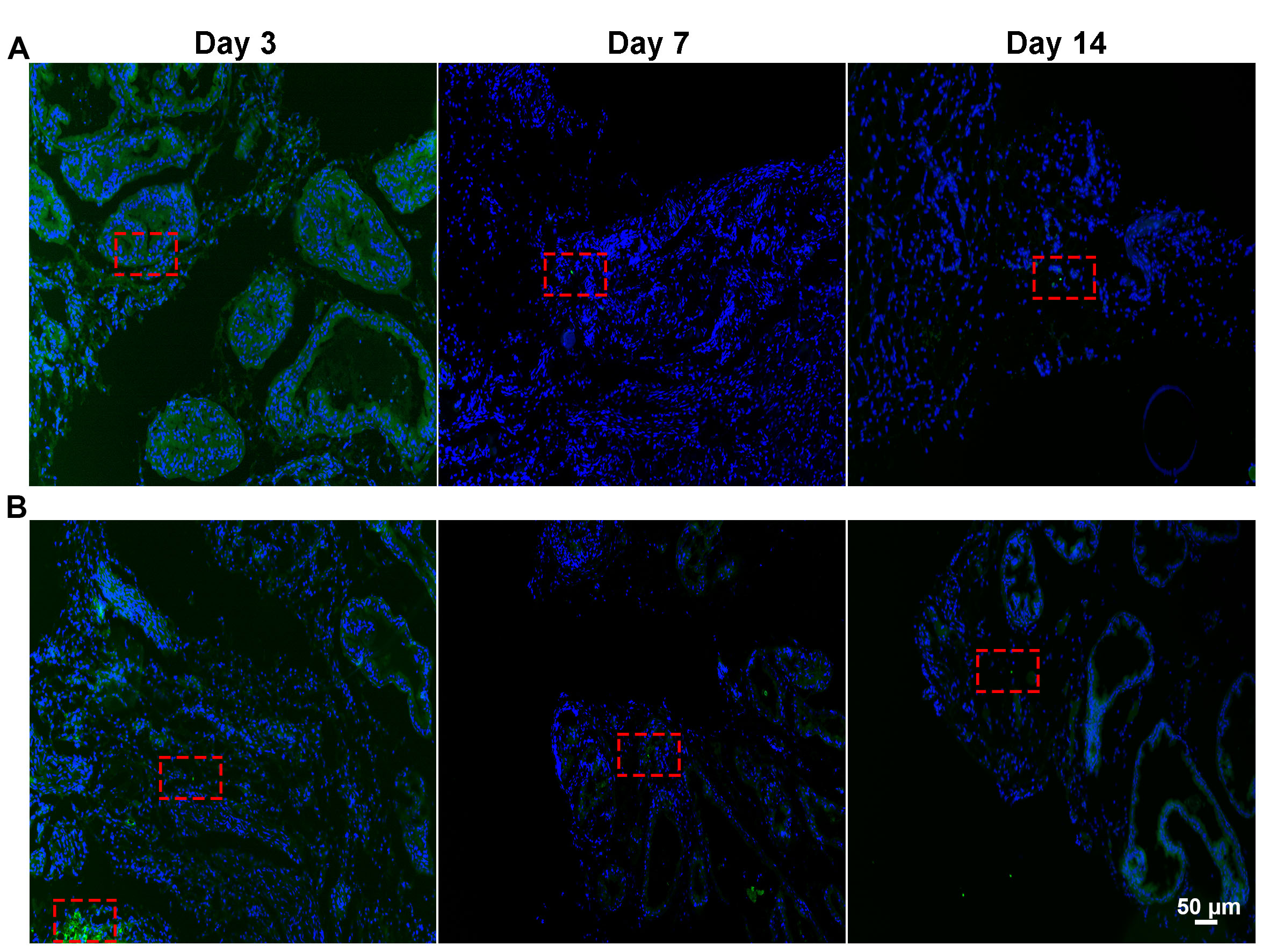

Supplement: Supplementary file 2 — Figure S2. PKH67 labelled cells in the MPG. A-B Labelled ADSCs and L-ADSCs could be detected in the MPG on Day 3, Day 7 and Day 14 after transplantation in rats with BCNI. ADSCs: adipose tissue-derived stem cells; L-ADSCs: lipopolysaccharide-preconditioned adipose tissue-derived stem cells; BCNI: bilateral cavernous nerve injury; MPG: major pelvic ganglion. [file 12610_2022_156_MOESM2_ESM.jpg]

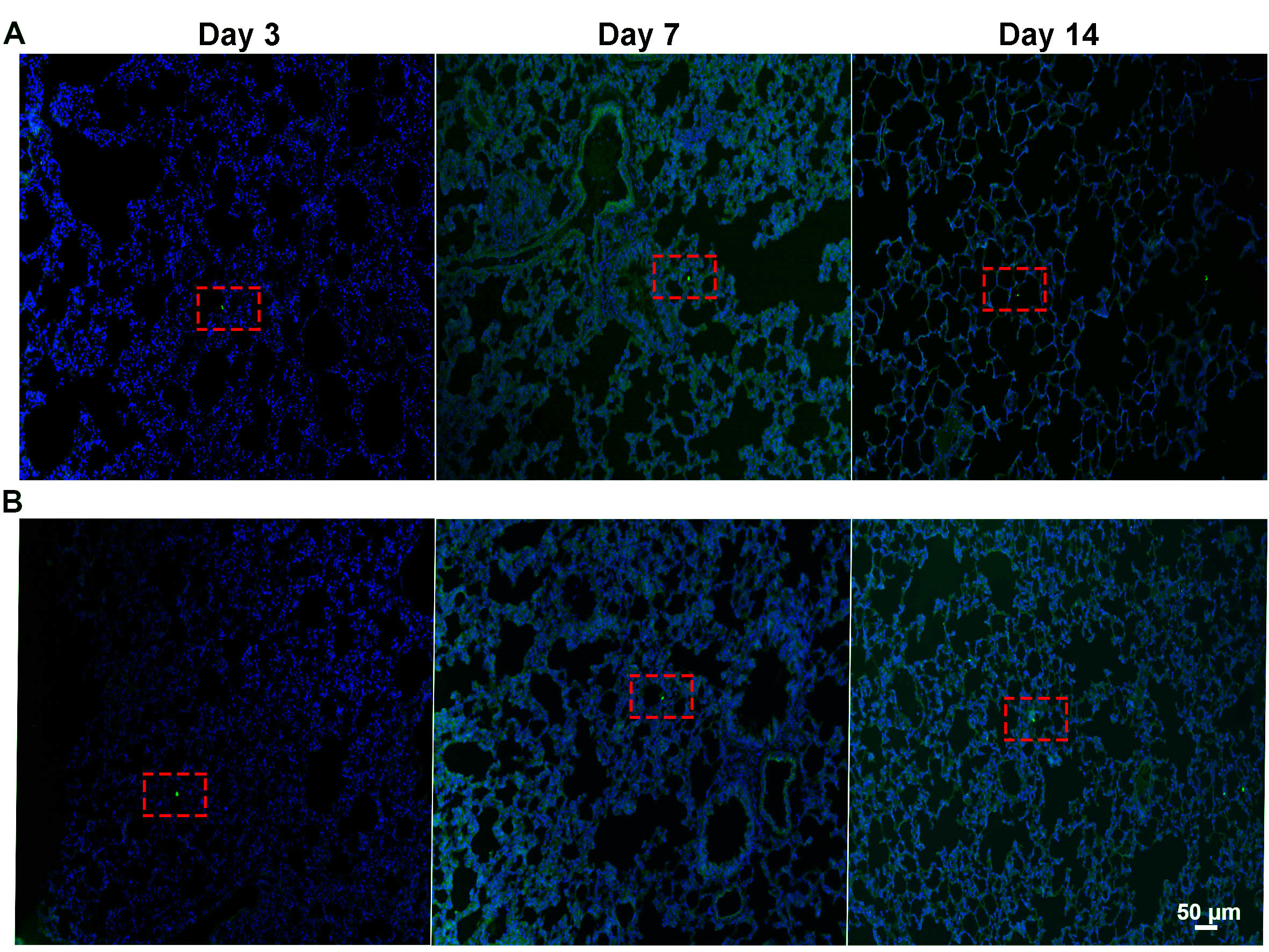

Supplement: Supplementary file 3 — Figure S3. PKH67 labelled cells in the lung. A-B Labelled ADSCs and L-ADSCs could be detected in the lung on Day 3, Day 7 and Day 14 after transplantation in rats with BCNI. ADSCs: adipose tissue-derived stem cells; L-ADSCs: lipopolysaccharide-preconditioned adipose tissue-derived stem cells; BCNI: bilateral cavernous nerve injury. [file 12610_2022_156_MOESM3_ESM.jpg]

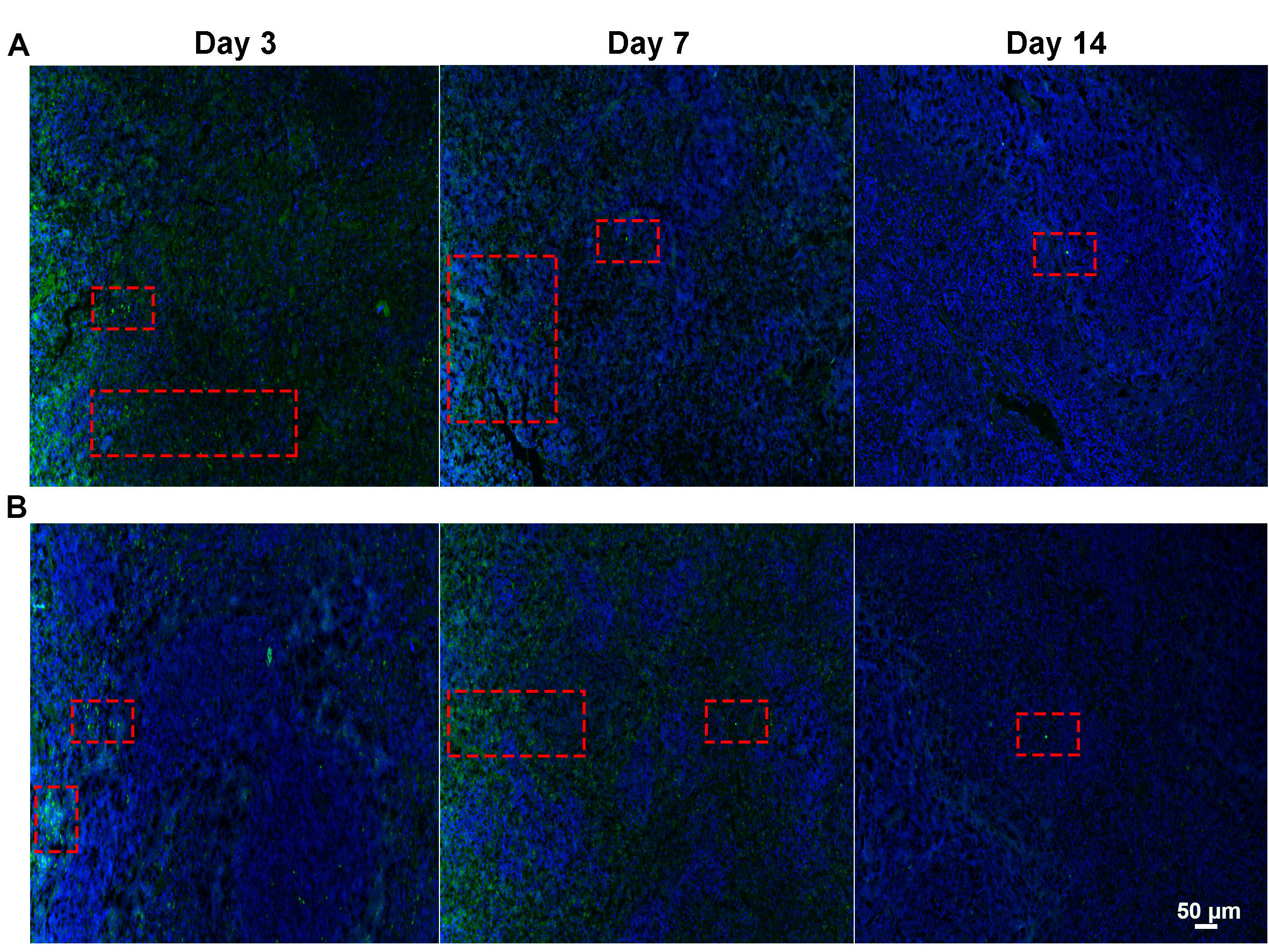

Supplement: Supplementary file 4 — Figure S4. PKH67 labelled cells in the spleen. A-B Labelled ADSCs and L-ADSCs could be detected in the spleen on Day 3, Day 7 and Day 14 after transplantation in rats with BCNI. ADSCs: adipose tissue-derived stem cells; L-ADSCs: lipopolysaccharide-preconditioned adipose tissue-derived stem cells; BCNI: bilateral cavernous nerve injury. [file 12610_2022_156_MOESM4_ESM.jpg]
